# Genetic Variants in *Caveolin-1* and *RhoA/ROCK1* Are Associated with Clear Cell Renal Cell Carcinoma Risk in a Chinese Population

**DOI:** 10.1371/journal.pone.0128771

**Published:** 2015-06-12

**Authors:** Ruizhe Zhao, Kang Liu, Zhengkai Huang, Jun Wang, Yongsheng Pan, Yuan Huang, Xiaheng Deng, Jinliang Liu, Chao Qin, Gong Cheng, Lixin Hua, Jie Li, Changjun Yin

**Affiliations:** State Key Laboratory of Reproductive Medicine, Department of Urology, First Affiliated Hospital of Nanjing Medical University, Nanjing 210029, China; Beijing University of Chemical Technology, CHINA

## Abstract

**Background:**

The *RhoA/ROCK* pathway and *Caveolin-1* (*Cav-1*) participate in the process of tumorigenesis in numerous types of cancer. Up-regulation of *RhoA/ROCK* and *Cav-1* expression is considered to be associated with the development and progression of clear cell renal cell carcinoma (ccRCC). We investigated the association between genetic variations of *RhoA/ROCK* and Cav-1 and the risk of ccRCC in the Chinese population.

**Methods:**

Between May 2004 and March 2014, a total of 1,248 clear cell renal cell carcinoma cases and 1,440 cancer-free controls were enrolled in this hospital-based case-control study. Nine SNPs in *RhoA/ROCK* and *Cav-1* were genotyped using the TaqMan assay.

**Result:**

We found two SNPs (*Cav-1* rs1049334 and *ROCK1* rs35996865) were significantly associated with the increasing risk of ccRCC (*P* = 0.002 and *P* < 0.001 respectively). The analysis of combined risk alleles revealed that patients with 2–4 risk alleles showed a more remarkable growth of ccRCC risk than the patients with 0–1 risk alleles(OR = 1.66, 95%CI = 1.31–2.11, *P* < 0.001). Younger subjects (*P* = 0.001, OR = 1.83, 95%CI = 1.30–2.57), higher weight subjects (*P* = 0.001, OR = 1.76, 95%CI = 1.25–2.47), female subjects (*P* = 0.007, OR = 1.75, 95% CI = 1.17–2.62), nonsmokers (*P* < 0.001, OR = 1.67, 95%CI = 1.26–2.23), drinkers (P = 0.025, OR = 1.75, 95% CI = 1.07–2.85), subjects with hypertension (*P* = 0.025, OR = 1.75, 95% CI = 1.07–2.85) and diabetes (*P* = 0.026, OR = 4.31, 95% CI = 1.19–15.62) showed a stronger association between the combined risk alleles and the risk of ccRCC by using the stratification analysis. Furthermore, we observed higher *Cav-1* mRNA levels in the presence of the rs1049334 A allele in normal renal tissues.

**Conclusion:**

Our results indicate that the two SNPs (*Cav-1* rs1049334 and *ROCK1* rs35996865) and genotypes with a combination of 2–4 risk alleles were associated with the risk of ccRCC. The functional SNP rs1049334 may affect the risk of ccRCC by altering the expression of Cav-1 and the relevance between the risk effects and the functional impact of this polymorphism needs further validation.

## Introduction

Renal cell carcinoma accounts for the majority, which is more than 80%, of the malignancy of renal and the clear cell type is the most common subtype. It is reported that clear cell renal cell carcinoma(ccRCC) has become the seventh most common cancer type for its steady increase annually, accounting for 270,000 newly diagnosed cases and estimated 116,000 cancer deaths [1]. In China, ccRCC is also a big public health issue because of high incidence and limited medical infrastructure and awareness.

As with most human cancers, the genesis of ccRCC is very complex, and the molecular mechanisms underlying disease occurrence are still largely unknown. It is reported that western people show higher morbidity compared with Chinese population, which may be a result of geographic, lifestyle and genetic variations. Studies associated with the risk factors of ccRCC demonstrated that smoking, drinking, HBP and high BMI might contribute to the tumorgenesis. However, only a small number of people who share the same risk factors suffer from this disease, indicating that genetic variation may be an assignable reason of the origin of tumor.

*RhoA* is a small GTP-binding protein that acts as a molecular switch that plays important roles in a diversity of cellular processes including motility, mitosis, proliferation and apoptosis. *RhoA* has a distinct set of effector kinases, including the *ROCK*, *CITRON*, and *PRK1*, all of which regulate cellular processes that contribute to tumorigenesis, invasion, and metastasis [2]. he *RhoA/ROCK* pathway participates in the process of angiogenesis in numerous types of cancer, by controlling the permeability, migration, proliferation, proliferation and morphogenesis of tumor cells [3]. *Cav-1* is secreted as a biologically active molecule of caveolae that promotes cell survival and angiogenesis within the tumor microenvironment, and is overexpressed in the metastatic and primary sites of several tumors. Previous studies demonstrated that *Cav-1* functioned as a positive effector of *RhoA* activation through the phosphorylation of cav-1 by Src kinases under certain circumstances [4]. The association between *RhoA/ROCK/Cav-1* and the genesis of tumor have raised increasing concerns.

To our knowledge, there is no report examining the association of single nucleotide polymorphisms (SNPs) in *ROCK1/RhoA* and Cav-1 and ccRCC risk. Considering that *ROCK1/RhoA* and *Cav-1* may play an important role in initiating the cancer, in the present study, we performed a hospital-based case–control study and selected and genotyped 9 tagging SNPs (tSNP) located in the functional regions of these genes to investigate the association of genetic polymorphisms of *ROCK1/RhoA* and *Cav-1* with ccRCC risk in Chinese population.

## Materials and Methods

### Ethics statement

The present study was approved by the Institutional Review Board of the Nanjing Medical University, Nanjing, China and each participant involved in this study gave a written informed consent prior to inclusion in the study.

### Study Population

In this study, 1248 patients with clear cell renal cell carcinoma and 1440 cancer-free age-matched controls were consecutively recruited from May 2004 to March 2014 at the First Affiliated Hospital of Nanjing Medical University. Patients with blood relationship or from the same region or the same families were excluded from this study in advance. All the diagnosis of clear cell renal cell carcinoma was established by pathological examination of samples resected by surgery and none of the patients had history of other cancers or relative therapy before. Each patient’s ccRCC classification and staging were according to the TNM staging system by American Joint Committee on Cancer (AJCC). The Furman scale was used to assess nuclear grade of ccRCC. The 1440 controls were people who visited for regular health examination in the outpatient departments and declared free of any cancers. They were recruited frequency matched to the cases on age (±5 years) and gender. A written informed consent was given to each patient and all of them approved to donate 5ml venous blood, restoring in a condition of -20°C anticoagulated by EDTA.

### SNP Selection

According to the CHB (i.e. Han Chinese in Beijing, China) data from HapMap (http://hapmap.ncbi.nlm.nih.gov/) and dbSNP (http://www.ncbi.nlm.nih.gov/projects/SNP/) and Haploview software 4.2 (http://www.broad-institute.org/haploview), tag-SNPs in *ROCK1/RhoA* and *Cav-1* via a pair-wise tag-SNP algorithm was selected based on correlation coefficient (r2) linkage disequilibrium. The screening criteria were (i) minor allele frequency > 5% in the Chinese population and (ii) r^2^ threshold > 0.8. Minor allele frequency(MAF) of all of these genes is more than 5% in the Han Chinese population. Finally, 3 SNPs in *Cav-1*(rs1049314, 1049337, rs1049334), 3 SNPs in *ROCK1* (rs8089974, rs35996865, rs11874761) and 3 SNPs in *RhoA* (rs2269736, rs2410, rs2625955) were included in this study.

### DNA extraction and Genotyping

Genomic DNA of each individual was isolated and purified from 500 ml EDTA-anticoagulated peripheral blood samples using a DNA extraction kit (Tiangen Biotech, Beijing, China) following the manufacturer’s instructions. Genotyping of the selected SNPs was conducted using the TaqMan SNP Genotyping Assay, which were performed on the 384-well LightCycler 480 Real Time PCR System (Roche, Penzberg, Germany). PCR was performed in a mixture containing 1.5μL SNP Genotyping Assay Mix, 1.5μL TaqMan Universal Master Mix, 1μL Dnase-free water and 1μL genomic DNA. The PCR conditions were 2 min at 50°C, 10 min at 95°C, followed by 40 cycles at 95°C for 15 sec and 60°C for 1 min. The LightCycler480 Software (Version 1.5.0) was used to automatically collect and analyze the data and to generate the genotype calls. The quality control was performed in each plate by four negative controls to ensure the genotyping accuracy. Regenotyping was conducted in approximately 10% of the samples and all the results were 100% concordant.

### Analysis of *Cav-1* and *ROCK1* expressions

To further assess the correlations between the *Cav-1* and *ROCK1* mRNA expressions and the polymorphisms of rs1049334 and rs35996865 *in vivo*, a total of 64 paratumor renal tissues adjacent to tumour containing 100% normal cells were obtained from patients. Total RNA was extracted using Trizol reagent (Invitrogen, Carlsbad, CA, USA) according to the manufacturer’s protocol. The 1.5-μg total RNA was reverse transcribed in a final volume of 20 μl using random primers under standard conditions using the PrimeScript RT Master Mix (Invitrogen). The quantitative real-time reverse transcription (RT-PCR) was conducted to measure the mRNA level of *Cav-1* and *ROCK1* on the Roche LightCycler 480 Real Time PCR System. The primers used for *Cav-1* were 5’- CGCCATTCTCTCTTTCCTGC-3’(forward) and 5’–AGACGGTGTGGACGTAGA- TG-3’(reverse) and for *ROCK1* were 5’- AAGAGAGTGATATTGAGCAGTT- GCG-3’(forward) and 5’—TTCCTCTATTTGGTACAGAAAGCCA-3’(reverse) for β-actin were 5’-ACTGGAACGGTGAAGGTGAC-3’(forward) and 5’-AGAGAA- GTGGGGTGGCTTTT-3’ (reverse). β-actin was used as an internal quantitative control, and each reaction was performed in triplicate. The qRT-PCR reaction included an initial denaturation step at 95 ° C for 10 min, followed by 40 cycles of 92 ° C for 15 s and 60 ° C for 1 min.

### Statistical Analysis

SPSS 16.0 (IL, CA, USA) was used for the statistical analysis. To assess the Hardy-Weinberg equilibrium of the genotype distribution, we performed a goodness-of-fit χ^2^ test. The differences in frequency distributions of epidemiological factors like lifestyles and personal health condition between ccRCC cases and controls were tested by using the t test for continuous variables and the χ^2^-test for categorical variables, respectively. Odds ratios (ORs) and the 95% confidence intervals (CIs) for the association between the polymorphisms and risk of ccRCC was analyzed by unconditional logistic regression, adjusting for age, smoking and drinking status, family history and other factors. Additionally, the false discovery rate (FDR) was used to adjust the *P* value for multiple comparisons, based on the Benjamini–Hochberg method. The associations were considered statistically significant when Bonferroni FDR-adjusted *P* values were <0.05. We used the Student’s t test or one-way analysis of variance (ANOVA) to explore associations between the two polymorphisms (rs35996865 and rs1049334) and the mRNA levels of ROCK1 and Cav-1. All statistical analyses were two-sided and *P* <0.05 was considered statistically significant.

## Results

### Characteristics and clinical features of all subjects

As is shown in **Table 1**, age (*P* = 0.602), gender (*P* = 0.068) and drinking status (*P* = 0.618) were matched between cases and controls. Nevertheless, more smokers (*P* <0.001), higher BMI level (*P* = 0.002), diabetics (*P* < 0.001) and hypertension patients (*P* <0.001) were observed in the ccRCC group compared with the controls. The prevalence of clinical stage I, II, III and IV were 821(65.8%), 243(19.5%), 90(7.2%) and 94(7.5%) in the 1248 ccRCC patients, and those of nuclear grade from I to IV were 240 (19.2%), 645 (51.7%), 277 (22.2%) and 86 (6.9%), respectively.

**Table 1:** Distribution of selected characteristics among the ccRCC cases and control subjects.

Characteristic	Cases(n = 1248)		Controls(n = 1440)		*P* *
	n	%	n	%	
Age (years)(Mean ± SD)	56.8±12.3		56.6±11.7		0.602
BMI (kg/m2) (Mean ± SD)	24.1±2.9		23.8±3.2		**0.002**
Gender					
Male	792	63.5	962	66.8	0.068
Female	456	36.5	478	33.2	
Smoking status					
Never	808	64.8	962	66.8	**<0.001**
Former	180	14.4	81	5.6	
Current	260	20.8	397	27.6	
Drinking status					
Never	908	72.8	1060	73.6	0.618
Ever	340	27.2	380	26.4	
Hypertension					
No	765	61.3	1071	74.4	**<0.001**
Yes	483	38.7	369	25.6	
Diabetes					
No	1087	87.1	1365	94.8	**<0.001**
Yes	161	12.9	75	5.2	
Clinical stage					
Ⅰ	821	65.8			
Ⅱ	243	19.5			
Ⅲ	90	7.2			
Ⅳ	94	7.5			
Grade					
Ⅰ	240	19.2			
Ⅱ	645	51.7			
Ⅲ	277	22.2			
Ⅳ	86	6.9			

*T-test for age and BMI distributions between the cases and controls; two-sided χ^2^ test for others selected variables between the cases and controls.

### Association between renal cell carcinoma risk and genetic polymorphisms of *Cav-1* and *ROCK1/RhoA*

A few genetic characteristics of the 9 selected tSNPs were presented in **Table 2**. The tSNP of rs1049337 in Cav-1 was excluded from further analysis because of the allele frequencies in control group not conforming to HWE (*P* <0.001). Genotype and allele distributions of the remaining 8 tSNPs in the patients and controls were detailed in **Table 3**. No significant differences in genotype and allele distributions of tSNPS in RhoA were observed between the cases and controls (rs2269736 *P* = 0.155, rs2410 *P* = 0.388, rs2625955 *P* = 0.595). We found two polymorphisms were significantly associated with ccRCC: rs1049334 in *Cav-1* and rs35996865 in *ROCK1* (*P =* 0.002 and *P <*0.001 respectively).

**Table 2:** The characteristics of the 9 tSNPs in *Cav-1* and *RhoA/ROCK1*.

Polymorphism	Alleles	Location	MAF	HWE*
rs2410	C>A	3'-UTR	0.467	0.494
rs11874761	G>A	5'-UTR	0.085	0.165
rs2625955	A>C	5'-neargene	0.427	0.979
rs35996865	T>G	5'-neargene	0.085	0.052
rs1049334	G>A	3'-UTR	0.159	0.057
rs1049337	T>C	3'-UTR	0.387	0.000
rs2269736	G>A	5'-UTR	0.317	0.718
rs8089974	T>G	5'-neargene	0.083	0.181
rs1049314	C>T	3'-UTR	0.169	0.832

*χ^2^ test was used to assess Hardy–Weinberg equilibrium (HWE) in controls

**Table 3:** The basic information of the genotyped polymorphisms in nine SNPs in the *RhoA/ROCK1* and *Cav-1* associated with the ccRCC risk.

Polymorphisms	cases(n = 1248)		controls(n = 1440)		*P* ^a^	FDR^b^	Adjusted OR (95% CI)^c^
	n	%	n	%			
**rs2410**							
TT	350	28.0	436	30.3	0.388		1.00 (reference)
GG	261	20.9	303	21.0			1.08(0.87–1.36)
GT	637	51.0	701	48.7			1.19(0.99–1.43)
GT+GG	898	72.0	1004	69.7	0.218		1.16(0.98–1.38)
T allele	1337	53.6	1573	54.6	0.442		1.05(0.94–1.18)
G allele	1159	46.4	1307	45.4			
**rs11874761**							
GG	1020	81.7	1158	80.4	0.668		1.00 (reference)
AA	9	0.7	10	0.7			0.87(0.34–2.24)
AG	219	17.5	272	18.9			0.92(0.75–1.13)
AG+AA	228	18.3	282	19.6	0.402		0.92(0.75–1.12)
G allele	2259	90.5	2588	89.9	0.436		0.90(0.80–1.17)
A allele	237	9.5	292	10.1			
**rs2625955**							
AA	480	38.5	581	40.3	0.595		1.00 (reference)
CC	175	14.0	192	13.3			1.06(0.83–1.36)
CA	593	47.5	667	46.3			1.05(0.88–1.24)
CA+CC	768	61.5	859	59.7	0.323		1.05(0.90–1.24)
A allele	1553	62.2	1829	63.5	0.336		1.02(0.87–1.21)
C allele	943	37.8	1051	36.5			
**rs35996865**							
TT	955	78.9	1157	80.3	**<0.001**		1.00 (reference)
GG	36	2.9	8	0.6			**5.41(2.47–11.83)**
GT	257	18.2	275	19.1			1.17(0.96–1.43)
GT+GG	293	21.1	283	19.7	**0.016**	**0.048**	**1.30(1.07–1.57)**
T allele	2167	88.0	2589	89.9	**<0.001**		**1.39(1.17–1.65)**
G allele	329	12.0	291	10.1			
**rs1049334**							
GG	765	61.3	968	66.5	**0.002**		1.00 (reference)
AA	77	6.2	60	5.1			**1.68(1.17–2.41)**
AG	406	32.5	412	28.3			**1.30(1.09–1.54)**
AG+AA	483	38.7	472	33.5	**0.001**	**0.003**	**1.35(1.14–1.59)**
G allele	1936	77.6	2348	80.7	**<0.001**		**1.32(1.15–1.51)**
A allele	560	22.4	532	19.3			
**rs1049337**							
TT	394	31.6	453	31.5	0.502		1.00 (reference)
CC	273	21.9	341	23.7			0.89(0.72–1.11)
CT	581	46.6	646	44.9			1.04(0.87–1.25)
CT+CC	854	68.4	987	68.5	0.967		0.99(0.84–1.17)
T allele	1369	54.8	1552	53.9	0.493		0.95(0.84–1.22)
C allele	1127	45.2	1328	46.1			
**rs2269736**							
GG	436	34.9	549	38.1	0.155		1.00 (reference)
AA	182	14.6	216	15.0			0.98(0.77–1.25)
AG	630	50.5	675	46.9			1.18(0.99–1.41)
AG+AA	812	65.1	891	61.9	0.092		1.13(0.96–1.33)
G allele	1502	60.2	1773	61.6	0.3		1.15(0.94–1.37)
A allele	994	39.8	1107	38.4			
**rs8089974**							
TT	1020	81.7	1160	80.6	0.663		1.00 (reference)
GG	10	0.8	10	0.7			0.95(0.38–2.37)
GT	218	17.5	270	18.8			0.93(0.76–1.14)
GT+GG	228	18.3	280	19.4	0.459		0.93(0.76–1.14)
T allele	2258	90.5	2590	89.9	0.52		0.90(0.71–1.09)
G allele	238	9.5	290	10.1			
**rs1049314**							
CC	1235	99.0	1424	98.9	0.999		1.00 (reference)
TT	0	0.0	0	0.0			
TC	13	1.0	16	1.1			0.91(0.42–1.96)
TC+TT	13	1.0	16	1.1	0.999		0.91(0.42–1.96)
C allele	2483	99.5	2864	99.4	0.999		0.90(0.42–1.93)
T allele	13	0.5	16	0.6			

a:Two-sided χ2 test for either genotype distributions or allele frequencies between the cases and controls.

b: Bonferroni FDR

c:Adjusted for age, BMI, gender, smoking status, drinking status and history of hypertension and diabetes in logistic regression model; 95% CI: 95% confidence interval

We found rs1049334 (G>A), which is located in *Cav-1*, was significantly associated with risk of ccRCC. Subjects with GA and AA genotypes had a significant increased ccRCC risk compared with those with GG (AA vs GG: adjusted OR = 1.68, 95% CI = 1.17–2.41; GA vs GG: adjusted OR = 1.30, 95% CI = 1.09–1.54). When combined the subjects with GA and AA genotypes, an increased risk of ccRCC was also observed in the combined group (adjusted OR = 1.33, 95% CI = 1.14–1.59). Alleles comparison showed similarly ccRCC risk rising (*P* < 0.001, OR = 1.32, 95%CI = 1.15–1.51). Another SNP (rs35996865) located in *ROCK1* was also found to be associated with ccRCC risk. For this SNP, significant association was observed in the subjects with GG genotype and dominant model (GG vs.TT, OR = 5.41, 95%CI = 2.47–11.83); GG+ GT vs.TT, OR = 1.30, 95%CI = 1.09–1.54). A similar result was observed when compared G alleles with T alleles (*P* < 0.001, OR = 1.39, 95%CI = 1.17–1.65).

### The analysis of combined polymorphisms and ccRCC risk

The two polymorphisms (rs1049334 and rs35996865) were combined based on the number of the risk alleles to explore the genetic power on the risk of ccRCC. As is shown in the **Table 4**, with the increasing of the number of the risk alleles, elevation of the ccRCC risk was observed and the difference was statistically significant. What’s more, when we divided the patients with two groups, patients with 2–4 risk alleles showed a more remarkable growth of ccRCC risk than the patients with 0–1 risk alleles(OR = 1.66, 95%CI = 1.31–2.11, *P* < 0.001) comparing with other group combinations. After that, stratification analysis indicated that the increased risk was more pronounced among younger subjects (*P* = 0.001, OR = 1.83, 95%CI = 1.30–2.57), higher weight subjects (*P* = 0.001, OR = 1.76, 95%CI = 1.25–2.47), female subjects (*P* = 0.007, OR = 1.75, 95% CI = 1.17–2.62), nonsmokers (*P* < 0.001, OR = 1.67, 95%CI = 1.26–2.23), drinkers (*P* = 0.025, OR = 1.75, 95% CI = 1.07–2.85), subjects with hypertension (*P* = 0.025, OR = 1.75, 95% CI = 1.07–2.85) and diabetes (*P* = 0.026, OR = 4.31, 95% CI = 1.19–15.62) **(Table 5)**. However, in patients with localized (stage I and II) or advanced stage (stage III and IV) and moderately (grade I and II) or poorly differentiated (grade III and IV) nuclear grade, no significant difference was observed (**S1 Table**).

**Table 4:** Analysis between combined risk alleles and ccRCC Susceptibility.

	cases(n = 1248)		controls(n = 1440)		*P* *	Adjusted OR (95% CI)^△^
	n	%	n	%		
Number of risk alleles						
0	584	46.8	776	53.9		1.00(reference)
1	474	38.0	520	36.1	**0.005**	**1.28(1.08–1.51)**
2	159	12.7	131	9.1	**<0.001**	**1.69(1.30–2.20)**
3	27	2.2	11	0.8	**0.001**	**3.41(1.66–7.02)**
4	4	0.3	2	0.1	**0.201**	3.07(0.55–17.17)
Recombined groups						
0–1	1058	84.8	1296	90.0	**<0.001**	1.00(reference)
2–4	190	15.2	144	10.0		**1.66(1.31–2.11)**

*Two-sided χ^2^ test for either genotype distributions or allele frequencies between the cases and controls.

△Adjusted for age, gender, body mass index, smoking status, drinking status, hypertension and diabetes in logistic regression model; 95% CI: 95% confidence interval.

**Table 5:** Association between *Cav-1* and *ROCK1* polymorphism and clinicopathologic characteristics of ccRCC.

	Risk allele					
	0–1		2–4		*P* *	Adjusted OR(95% CI) ^△^
	n	%	n	%		
Clinical stage						
I + II	896	84.2	168	15.8	0.221	1.00(reference)
III + IV	162	88.0	22	12.0		0.74(0.45–1.26)
Grade						
I + II	745	84.2	140	15.8	0.386	1.00(reference)
III + IV	313	86.2	50	13.8		0.93(0.64–1.35)

*Two-sided χ^2^ test for number of alleles in cases and controls.

△Adjusted for age, gender, body mass index, smoking status, drinking status, hypertension and diabetes in logistic regression model; 95% CI: 95% confidence interval

### Associations between *Cav-1* rs1049334 and *ROCK1* rs35996865 and the expression levels of corresponding mRNA

**Fig 1:**
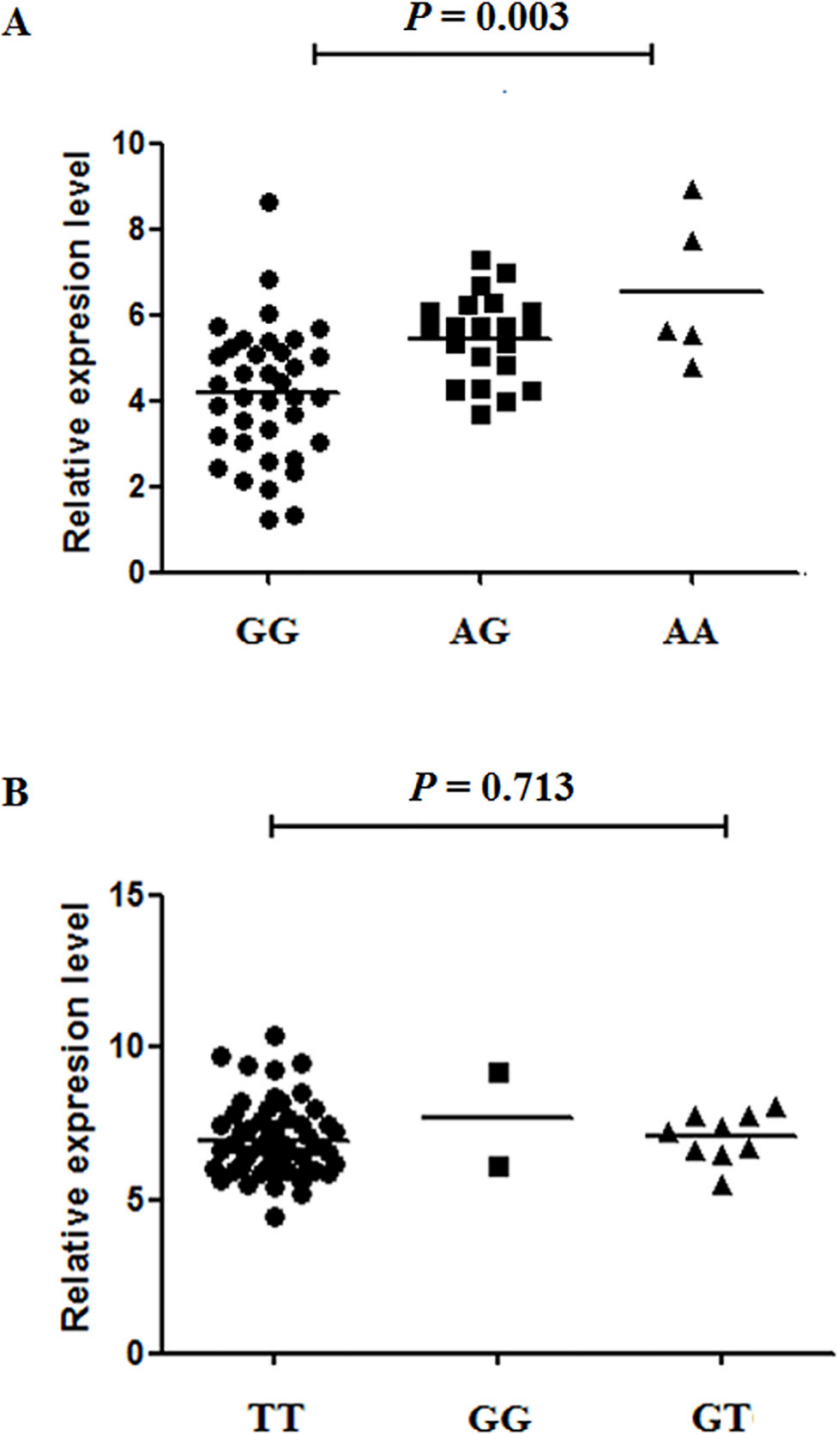
Expression of *Cav-1* in cancer adjacent normal renal tissues. (A), Samples with rs1049334 AG or AA genotypes were associated with higher expression levels of *Cav-1* mRNA compared with samples with GG genotypes in normal tissues of ccRCC patients (*P* = 0.003). (B), No significant differences in *ROCK1* expression among different *ROCK1* rs35996865 genotypes were observed (*P* = 0.713).

In our study, 64 normal tumor-adjacent tissue samples were obtained to assess the associations between these two polymorphisms and the expression of *Cav-1* and *ROCK1* (**Fig 1**). According to the results, subjects with rs1049334 AA or AG genotypes had higher expression levels of *Cav-1* compared with those with GG genotypes (*P* = 0.003). However, the *ROCK1* expression level in patients carrying three types of rs35996865 genotypes was similar (*P* = 0.713), suggesting that the influence of rs3599686 polymorphism on gene expression is weak.

### Stratification analyses between *Cav-1* rs1049334 and risk of ccRCC

A stratification analysis was then evaluated by the age, gender, BMI, smoking status, drinking status, history of hypertension and diabetes (**S2 Table**). As a result, we found that the increased risk of ccRCC was more remarkable between older subjects (*P* = 0.005, OR = 1.44, 95%CI = 1.14–1.81), higher weight subjects (*P* = 0.015, OR = 1.37, 95%CI = 1.08–1.74), male subjects (*P* = 0.021, OR = 1.37, 95% CI = 1.11–1.69), nonsmokers (*P* = 0.002, OR = 1.38, 95%CI = 1.13–1.68), nondrinkers (*P* = 0.006, OR = 1.38, 95%CI = 1.14–1.66), subjects without hypertension (*P* = 0.006, OR = 1.34, 95% CI = 1.10–1.64) and without diabetes (*P* = 0.001, OR = 1.35, 95% CI = 1.14–1.60). Besides, no significant associations between Cav1 rs1049334 polymorphism and clinical stage or tumor grade of ccRCC patients were observed (**Table 6**).

**Table 6:** The association of *Cav1* rs1049334 polymorphism and clinicopathologic characteristics of ccRCC patients.

	GG		AG/AA		*P* *	Adjusted OR (95% CI) ^△^
	n	%	n	%		
Clinical Stage						
Ⅰ	498	49.7	323	49.8	0.764	1.00(reference)
Ⅱ	386	38.5	259	40.0		0.93(0.68–1.27)
Ⅲ	57	5.7	33	5.1		0.84(0.52–1.36)
Ⅳ	61	6.1	33	5.1		0.90(0.54–1.52)
Grade						
Ⅰ	154	20.1	86	17.8	0.408	1.00(reference)
Ⅱ	386	50.5	259	53.6		1.27(0.93–1.74)
Ⅲ	167	21.8	110	22.8		1.31(0.88–1.95)
Ⅳ	58	7.6	28	5.8		0.95(0.52–1.73)

*Two-sided χ^2^ test for number of alleles in cases and controls.

△Adjusted for age, BMI, gender, smoking status, drinking status and history of hypertension and diabetes in logistic regression model; 95% CI: 95% confidence interval

## Discussion

*Caveolin-1* (*Cav1*) is a principal functional constituent of caveolae, which are invaginated plasma membrane microdomains and function as a regulator of signal transduction events and cytoskeletal dynamics [5,6]. Glenney first reported that *Caveolin-1* was a novel substrate for the src kinase oncogene in virally transformed fibroblasts by binding to several key proteins [7]. *Cav-1* has been proved to modulate multiple cancer-associated processes including cellular transformation, tumor growth, cell migration and metastasis, cell death and survival, multidrug resistance and angiogenesis in a number of signaling pathways [8,9]. Collective evidence from researches indicated that the elevated level of *Cav-1* was involved in some unfavorable clinical characteristics like larger size, higher grade and stage, resistance to conventional therapies and poor prognosis of various types of malignancy in several organs including colon, liver, stomach, prostate, breast, lung, brain and kidney [10–20]. Although the role of *Cav-1* plays in cancer is controversial [12], it is widely confirmed that overexpression of *Cav-1* in renal cell carcinoma is associated with poor disease-free survival and metastasis [21–23]. Study in genetic variants revealed a significant association between *Cav-1* polymorphisms and ccRCC susceptibility [24].

*RhoA*, which is a predominant member of the well-known *Ras* superfamily of small guanosine triphosphatases (GTPases), is a small G protein that can exhibit intrinsic GTPases activities that can function as a molecular switch in cellular processes including cytoskeletal dynamics, migration, vesicle trafficking, cell proliferation, apoptosis and transcription [25–27]. A number of studies have shown that *RhoA* had abilities to control cancer metastasis and progression [28–30] and the expression of *RhoA* was up-regulated in several common malignancies including gastric, pancreatic and breast cancer [31–33]. *Rho*-associated coiled-coil forming kinase, which is often referred to as *ROCK*, is a major effector in the *Rho* signaling way. *ROCK1* is one of the *ROCK* family and plays a critical role in mediating the effects of *RhoA*, activated by binding to the Active (GTP-loaded) *Rho* [34,35]. The *Rho/Rho*-kinase pathway plays an important role in various cellular functions and is involved in several proinvasive pathways including *src*[36]. Accumulating evidences have shown that the interaction between *Caveolin-1* and *Rho*-GTPases could regulate metastasis by controlling the activation of src in malignancies [37,38]. However, study on the association between genetic variants in *Cav-1* with *RhoA/ROCK* and susceptibility of ccRCC is insufficient.

In our study, we genotyped nine polymorphisms in *RhoA/ROCK1* and *Cav-1* to explored the association between *RhoA/ROCK1* and *Cav-1* genetic variants and ccRCC susceptibility in Chinese population. The *Cav-1* rs1049334 and the *ROCK1* rs35996865 significantly differed between ccRCC patients and control participants, indicating that the risk of ccRCC is increased in participants with the A allele of rs1049334 and G allele of rs35996865. The further analysis of the combining risk alleles showed that the group of patients with 2–4 risk alleles was more susceptible to ccRCC compared with those with 0–1 risk alleles. Environmental and epidemiological factor also had certain effects on the risk of ccRCC according to our stratification analyses in combination alleles and *Cav-1* rs1049334. Age, BMI, gender, smoking and drinking status, the history of HBP and diabetes are all related with the ccRCC susceptibility, implying that the interaction of the environment, hereditary background and genetic variants may be a complex system contributed to the occurrence of ccRCC.

Additionally, increasing *Cav-1* mRNA level was found in individuals who carried the rs1049334 A allele in the preliminary functional analysis of the variant. Interestingly, stratification analyses of the association between the rs1049334 and the risk of ccRCC revealed a little different in history of hypertension and history of diabetes compared with that of risk alleles. We supposed that the role of *RhoA/ROCK1* in influencing the level of plasma glucose and vascular contractility may contribute to this procedure [39–41]. No significant association between the polymorphisms and clinicopathological characteristics of ccRCC was observed, we surmise that the effect of rs1049334 on the expression of *Cav-1* is not power enough to influence the disease progression.

Limitations should not be ignored in the present study. First, the sample is not large enough that bias there may exist in the analyzing of very low-penetrance SNPs and reduce the statistical power of combined analysis and stratification. Second, selection bias of subjects associated with a particular genotype could not be eliminated because our case-control study is hospital-based. However in present study, except for rs1049337, the rest 8 tSNPs all conformed to HWE. The selection bias might not be substantial because the distribution of genotypes was all similar to the Hapmap database of Chinese population.

In conclusion, we investigated an association between *Cav-1* and *RhoA/ROCK1* polymorphisms and susceptibility, clinical characteristics in a large sample population of ccRCC patients. *Cav-1* rs1049334 (G>A) and *ROCK1* rs35996865 (T>G) were significantly associated with the elevated risk of ccRCC in Chinese population, and the combination of risk alleles indicated a positive influence on the ccRCC risk. Furthermore, functional polymorphism in *Cav-1* rs1049334 (G>A) altered *Cav-1* expression, which may contribute to the genesis of ccRCC. Further functional investigations are expected to confirm our results.

## Supporting Information

S1 Table Stratification analysis of the variant numbers of genotypes by selected variables in ccRCC patients and controls (DOCX)

S2 Table Stratification analyses between the Cav1 rs1049334 polymorphisms and risk of clear cell renal cell carcinoma (DOCX)
