# Supplementary material for: Genetic Variants in Caveolin-1 and RhoA/ROCK1 Are Associated with Clear Cell Renal Cell Carcinoma Risk in a Chinese Population
Source: PLoS One. 2015 Jun 12;10(6):e0128771. doi: 10.1371/journal.pone.0128771 (PMC4467078; doi:10.1371/journal.pone.0128771)
Supplement: S1 Table — (DOCX) [file pone.0128771.s001.docx]

| S1 Table. Stratification analysis of the variant numbers of genotypes by selected variables in ccRCC patients and controls | | | | | | | | | | | | | |
| --- | --- | --- | --- | --- | --- | --- | --- | --- | --- | --- | --- | --- | --- |
|  | cases（n=1248） | | | | |  | controls（n=1440） | | | | | *P** | Adjusted OR (95% CI) ^△^ |
|  | Number of risk alleles | | | | |  | Number of risk alleles | | | | |  |  |
|  | 0-1 | |  | 2-4 | |  | 0-1 | |  | 2-4 | |  |  |
|  | n | % |  | n | % |  | n | % |  | n | % |  |  |
| Total | 1058 |  |  | 190 |  |  | 1296 |  |  | 144 |  | <0.001 | **1.66(1.31–2.11)** |
| Age(Years) |  |  |  |  |  |  |  |  |  |  |  |  |  |
| ≤57 | 543 |  |  | 91 |  |  | 736 |  |  | 70 |  | 0.001 | **1.83(1.30–2.57)** |
| >57 | 515 |  |  | 99 |  |  | 560 |  |  | 74 |  | 0.013 | **1.53(1.10–2.14)** |
| BMI |  |  |  |  |  |  |  |  |  |  |  |  |  |
| ≤24 | 531 |  |  | 97 |  |  | 690 |  |  | 69 |  | 0.009 | **1.57(1.12–2.20)** |
| >24 | 527 |  |  | 93 |  |  | 606 |  |  | 75 |  | 0.001 | **1.76(1.25–2.47)** |
| Gender |  |  |  |  |  |  |  |  |  |  |  |  |  |
| Male | 675 | 85.2 |  | 117 | 14.8 |  | 863 | 89.7 |  | 99 | 10.3 | 0.003 | **1.57(1.17–2.12)** |
| Female | 383 | 84.0 |  | 73 | 16.0 |  | 433 | 90.6 |  | 45 | 9.4 | 0.007 | **1.75(1.17–2.62)** |
| Smoking status |  |  |  |  |  |  |  |  |  |  |  |  |  |
| Never | 682 | 84.4 |  | 126 | 15.6 |  | 863 | 89.7 |  | 99 | 10.3 | <0.001 | **1.67(1.26–2.23)** |
| Ever | 150 | 83.3 |  | 30 | 16.7 |  | 70 | 86.4 |  | 11 | 13.6 | 0.247 | 1.63(0.71–3.75) |
| Current | 226 | 86.9 |  | 34 | 13.1 |  | 363 | 91.4 |  | 34 | 8.6 | 0.076 | 1.62(0.95–2.75) |
| Drinking status |  |  |  |  |  |  |  |  |  |  |  |  |  |
| Never | 768 | 84.6 |  | 140 | 15.4 |  | 953 | 89.9 |  | 107 | 10.1 | <0.001 | **1.65(1.25–2.17)** |
| Ever | 290 | 85.3 |  | 50 | 14.7 |  | 343 | 90.3 |  | 37 | 9.7 | 0.025 | **1.75(1.07–2.85)** |
| HBP |  |  |  |  |  |  |  |  |  |  |  |  |  |
| No | 648 | 84.7 |  | 117 | 15.3 |  | 963 | 89.9 |  | 108 | 10.1 | 0.001 | **1.60(1.20–2.13)** |
| Yes | 410 | 84.9 |  | 73 | 15.1 |  | 333 | 90.2 |  | 36 | 9.8 | 0.027 | **1.65(1.06–2.56)** |
| Diabetes |  |  |  |  |  |  |  |  |  |  |  |  |  |
| No | 919 | 84.5 |  | 168 | 15.5 |  | 1224 | 89.7 |  | 141 | 10.3 | <0.001 | **1.57(1.23–2.01)** |
| Yes | 139 | 86.3 |  | 22 | 13.7 |  | 72 | 96.0 |  | 3 | 4.0 | 0.026 | **4.31(1.19–15.62)** |

*Two-sided χ2 test for number of alleles in cases and controls.

△Adjusted for age, BMI, gender, smoking status, drinking status and history of hypertension and diabetes in logistic regression model; 95% CI: 95% confidence interval
