# Supplementary material for: Genetic Variants in Caveolin-1 and RhoA/ROCK1 Are Associated with Clear Cell Renal Cell Carcinoma Risk in a Chinese Population
Source: PLoS One. 2015 Jun 12;10(6):e0128771. doi: 10.1371/journal.pone.0128771 (PMC4467078; doi:10.1371/journal.pone.0128771)
Supplement: S2 Table — (DOCX) [file pone.0128771.s002.docx]

| S2 Table.Stratification analyses between the Cav1 rs1049334 polymorphisms and risk of clear cell renal cell carcinoma | | | | | | | |
| --- | --- | --- | --- | --- | --- | --- | --- |
|  | TT | |  | TA+AA | | *P** | Adjusted OR (95% CI) ^△^ |
|  | case（n,%） | control（n,%） |  | case（n,%） | control（n,%） |  |  |
| Age |  |  |  |  |  |  |  |
| ≤57 | 388 | 551 |  | 246 | 255 | 0.005 | **1.44(1.14–1.81)** |
| >57 | 377 | 417 |  | 237 | 217 | 0.112 | 1.27(0.99–1.61) |
| BMI |  |  |  |  |  |  |  |
| ≤24 | 385 | 513 |  | 239 | 252 | 0.042 | **1.31(1.04–1.64)** |
| >24 | 380 | 455 |  | 244 | 220 | 0.015 | **1.37(1.08–1.74)** |
| Gender |  |  |  |  |  |  |  |
| Male | 491 | 648 |  | 301 | 314 | 0.021 | **1.37(1.11–1.69)** |
| Female | 274 | 320 |  | 182 | 158 | 0.03 | 1.27(0.97–1.67) |
| Smoking status |  |  |  |  |  |  |  |
| Never | 490 | 651 |  | 318 | 311 | 0.002 | **1.38(1.13–1.68)** |
| Former | 113 | 57 |  | 67 | 24 | 0.263 | 1.49(0.80–2.77) |
| Current | 162 | 260 |  | 98 | 137 | 0.407 | 1.16(0.82–1.63) |
| Drinking status |  |  |  |  |  |  |  |
| Never | 555 | 720 |  | 353 | 340 | 0.002 | **1.38(1.14–1.66)** |
| Ever | 210 | 248 |  | 130 | 132 | 0.352 | 1.31(0.94–1.82) |
| HBP |  |  |  |  |  |  |  |
| No | 465 | 719 |  | 300 | 352 | 0.006 | **1.34(1.10–1.64)** |
| Yes | 300 | 249 |  | 183 | 120 | 0.112 | 1.23(0.91–1.66) |
| Diabetes |  |  |  |  |  |  |  |
| No | 657 | 915 |  | 430 | 450 | 0.001 | **1.35(1.14–1.60)** |
| Yes | 108 | 53 |  | 53 | 22 | 0.653 | 1.26(0.66–2.39) |

*T-test for age and BMI distributions between the cases and controls; two-sided χ2 test for others selected variables between the cases and controls.

△Adjusted for age, BMI, gender, smoking status, drinking status and history of hypertension and diabetes in logistic regression model; 95% CI: 95% confidence interval
